# Ang-1, Ang-2, and Tie2 are diagnostic biomarkers for Henoch-Schönlein purpura and pediatric-onset systemic lupus erythematous

**DOI:** 10.1515/biol-2022-0812

**Published:** 2024-02-08

**Authors:** Lishan Jia, Xiaozhong Li, Jiayun Shen, Yan Teng, Baoqin Zhang, Min Zhang, Yueqin Gu, Hong Xu

**Affiliations:** Department of Pediatrics, Taicang Affiliated Hospital of Soochow University, The First People’s Hospital of Taicang, No. 58 Changsheng South Road, Taicang City, Jiangsu Province, 215400, China; Department of Nephrology and Immunology, Children’s Hospital of Soochow University, No. 303 Jingde Road, Gusu District, Suzhou City, Jiangsu Province, 215003, China; Department of Nephrology, Children’s Hospital of Fudan University, No. 399 Wanyuan Road, Minhang District, Shanghai City, 201102, China

**Keywords:** Henoch-Schönlein purpura, systemic lupus erythematous, angiopoietins, Tie2 immunoglobulin A vasculitis, autoimmune diseases

## Abstract

Henoch-Schönlein purpura (HSP) and pediatric-onset systemic lupus erythematosus (pSLE) are closely associated with vasculitis and vascular diseases. This study aimed to investigate the clinical diagnostic values of Ang-1, Ang-2, and Tie2 for HSP and pSLE. We surveyed 82 HSP patients, 34 pSLE patients, and 10 healthy children. The expression levels of Ang-1, Ang-2, and Tie2 in the serum and urine were assessed using enzyme-linked immunosorbent assay. The diagnostic values of Ang-1, Ang-2, and Tie2 for HSP and pSLE were evaluated using receiver operating characteristic curve analysis. The results revealed that the serum and urine expression levels of Ang-2 and Tie2 were significantly elevated in HSP and pSLE patients, whereas the Ang-1/Ang-2 values were reduced. Additionally, Ang-1 was highly expressed in the serum and urine of HSP patients and in the serum of pSLE patients. Ang-1, Ang-2, and Tie2 showed differential expression in various types of HSP and pSLE compared with their expression in healthy controls. In summary, Ang-1, Ang-2, and Tie2 can serve as biomarkers for HSP and pSLE. Moreover, Ang-1/Ang-2 values are reduced in HSP and pSLE patients. Ang-1, Ang-2, and Tie2 can be used as biomarkers for HSP and pSLE.

## Graphical abstract

**Figure:**
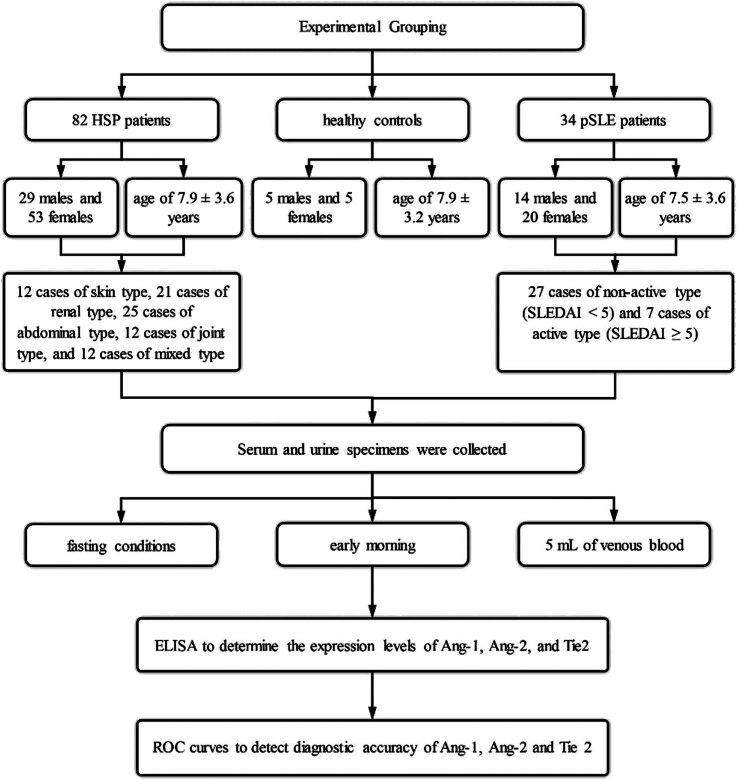

## 1 Introduction

Henoch-Schönlein purpura (HSP), also known as immunoglobulin A vasculitis, is characterized by IgA-dominated immune deposits in vessel walls [1]. HSP is the most common form of vasculitis in children, impacting 8–20 children per 100,000 annually. Among pediatric patients, 75–90% are younger than 10 years [2]. The clinical manifestations of HSP include palpable purpura, arthritis, abdominal pain, and renal involvement, which may progress to chronic kidney disease in severe cases [3,4]. Currently, the criteria for diagnosing children with HSP are mainly based on IgA deposits and kidney and joint skin involvement [5,6]. Rare complications associated with HSP can lead to misdiagnoses and delays in treatment, potentially resulting in poorer prognoses [7]. Therefore, it is crucial to explore diagnostic biomarkers for children with HSP.

Systemic lupus erythematosus (SLE) is an autoimmune disease associated with potential extensive angiopathy and vasculitis [8]. Approximately 11–36% of SLE patients present symptoms of vasculitis, which may lead to further visceral lesions, mesenteric vasculitis, pulmonary hemorrhage, and neuritis [9]. Pediatric-onset SLE (pSLE) accounts for approximately 10–20% of all SLE cases [10]. pSLE is characterized by its multisystemic and inflammatory nature [11]. It is more aggressive than adult-onset SLE, with greater organ and system involvement and imparting a higher drug burden, ultimately leading to high mortality rates and healthcare burden [12–15]. Moreover, pSLE-induced organ damage is significantly associated with longer disease duration, severe fatigue, and poorer quality of life [16]. pSLE is primarily diagnosed on the basis of antinuclear antibodies, anti-double stranded DNA antibodies, complement levels (C3, C4, CH50), and imaging studies [17–19]. However, these parameters lack sufficient sensitivity and specificity. Therefore, novel, specific, and safe biomarkers are urgently required to predict and detect pSLE.

The families of endothelial cell-specific receptor tyrosine kinases and their corresponding ligands/growth factors, including angiopoietins (Ang), ephrins, and vascular endothelial growth factors (VEGFs), are key contributors to vascular development, remodeling, and regeneration [20]. The Ang/Tie pathway is crucial for regulating vascular stability, angiogenesis, and the inflammatory response [21]. Ang-1 activates the Tie2 receptors, thereby promoting vascular stability, maturation, and integrity. Tie2 is a tyrosine kinase receptor expressed on the surface of vascular endothelial cells. Ang-2, an Ang-1 antagonist, competes to bind to Tie2 receptors [22]. The Ang/Tie2 signaling pathway is associated with multiple diseases. Ang-1/Tie2 signaling increases vascular access to tumor cells, promoting tumor dissemination and metastasis through vasodilation [23]. Purkinje cell dendritic development is autonomously regulated by the Ang-Tie2 pathway [24]. In ischemic diseases such as diabetic retinopathy, the upregulation of Ang-2 inactivates Tie-2, leading to vascular leakage, pericyte loss, and inflammation [25]. A cross-sectional analysis revealed a significant correlation between Tie2/Ang levels and the characteristics of circulating immune cells in well-differentiated neuroendocrine gastroenteropancreatic tumors [26]. Additionally, Tie2 levels in the serum of individuals with active pSLE were substantially higher than in those with an inactive condition [8]. However, the expression levels of Ang-1, Ang-2, and Tie2 in the urine and their diagnostic value in pSLE patients remain unclear. Furthermore, few studies have reported on the Ang/Tie2 pathway in HSP.

This study aimed to investigate the expression levels and diagnostic value of Ang/Tie2 in the serum and urine of HSP and pSLE patients. Initially, we assessed the levels of Ang-1, Ang-2, and Tie2 in both serum and urine using enzyme-linked immunosorbent assay (ELISA), revealing distinct expressions compared with those in healthy controls. Subsequently, we delved into the expression patterns of Ang-1, Ang-2, and Tie2 in patients with different types of HSP and pSLE. Finally, through receiver operating characteristic (ROC) curve analysis, we identified that Ang-1, Ang-2, and Tie2 could serve as diagnostic biomarkers in both serum and urine among HSP and pSLE patients. Our findings may identify diagnostic biomarkers and therapeutic targets for the treatment of HSP and pSLE patients.

## 2 Methods

### 2.1 Patients

Eighty-two HSP patients were recruited from the First People’s Hospital of Taicang, affiliated with Soochow University, the Children’s Hospital of Fudan University, and the Children’s Hospital of Soochow University, between July 2020 and July 2022. Additionally, 34 pSLE patients from the First People’s Hospital of Taicang, the Children’s Hospital of Fudan University, and the Children’s Hospital of Soochow University were enrolled. Ten healthy controls were enrolled from the Children’s Hospital of Fudan University and the Children’s Hospital of Soochow University during the same time period. The inclusion criteria were (1) patients with clinically confirmed HSP and pSLE, (2) all indicators within the normal ranges, (3) no other complications, (4) age ≤14 years, and (5) the patients’ families provided informed consent to their enrolment in the study. The exclusion criteria were (1) the presence of chronic infection with a history of serious infection in the last 2 months, (2) the combination of serious systemic, liver and kidney, and gastrointestinal diseases, (3) the presence of multiple autoimmune diseases, and (4) incomplete clinical data. The HSP, pSLE, and control groups did not differ significantly in terms of sex and age (*p* > 0.05). The study was approved by the ethics committee of the First People’s Hospital of Taicang, affiliated with Soochow University (Ethical number: KY-2020-204), and the sampling of all participants was approved by the participants’ families.


**Informed consent:** Informed consent has been obtained from all individuals included in this study.
**Ethical approval:** The research related to human use has been complied with all the relevant national regulations, institutional policies and in accordance with the tenets of the Helsinki Declaration, and has been approved by the ethics committee of the First People’s Hospital of Taicang, affiliated with Soochow University (Ethical number: KY-2020-204).

### 2.2 Determining the Ang-1, Ang-2, and Tie2 levels

Serum and urine specimens were collected from 82 HSP patients, 34 pSLE patients, and 10 healthy individuals. Specifically, a total of 5 mL of venous blood was collected from each participant in the early morning under fasting conditions and placed in a serum separator tube. The serum was then centrifuged at 4°C (3,500 rpm/10 min), and the upper layer was transferred into 1.5 mL centrifuge tubes for storage at −80°C. Early morning mid-stream urine samples were also collected, centrifuged to remove large cell fractions (1,500 rpm/5 min), and the supernatant was divided into 1.5 mL centrifuge tubes and stored at −80°C. The centrifuge tubes were removed from storage 30 min before the assay and allowed to return to room temperature. The expression levels of Ang-1, Ang-2, and Tie2 in the serum and urine were determined using ELISAs [27] (Esebio, Shanghai, China), according to the manufacturer’s instructions.

### 2.3 Statistical analysis

SPSS (v. 26.0, Chicago, IL) software was used for data analyses. Measures obeying normal distribution were expressed as mean ± standard deviation (*x̅* ± s) and the Tukey’s test-corrected one-way analysis of variance was used for multiple group comparisons. Non-normally distributed measures were expressed as medians (quartiles, M [P25, P75]), and the Wilcoxon rank-sum test was used to analyze non-parametric data. *p* < 0.05 was considered statistically significant. ROC curve analysis was used to identify optimal areas under the curve (AUCs) to determine diagnostic value.

## 3 Results

### 3.1 Demographic and clinical characteristics

The 82 HSP patients included 12 with dermatological (14.6%), 21 with renal (25.6%), 25 with abdominal (30.5%), 12 with joint-type (14.6%), and 12 with mixed-type (14.6%) HSP, with a mean age of 7.9 ± 3.6 years. The HSP patients comprised 29 male and 53 female children and the male:female ratio was 1:5.5. According to the SLE Disease Activity Index (SLEDAI) scores, the 34 pSLE patients comprised 27 with non-active (SLEDAI < 5; 79.4%) and 7 with active SLE (SLEDAI ≥ 5; 20.6%). This group included 14 male (41.2%) and 20 female (58.8%) children, with an average age of 7.5 ± 3.6 years. Ten healthy individuals were also enrolled as a control group, comprising five male (50%) and five female (50%) children, with a mean age of 7.9 ± 3.2 years.

### 3.2 Ang-1/Ang-2 values are reduced in HSP and pSLE patients

**Figure 1:**
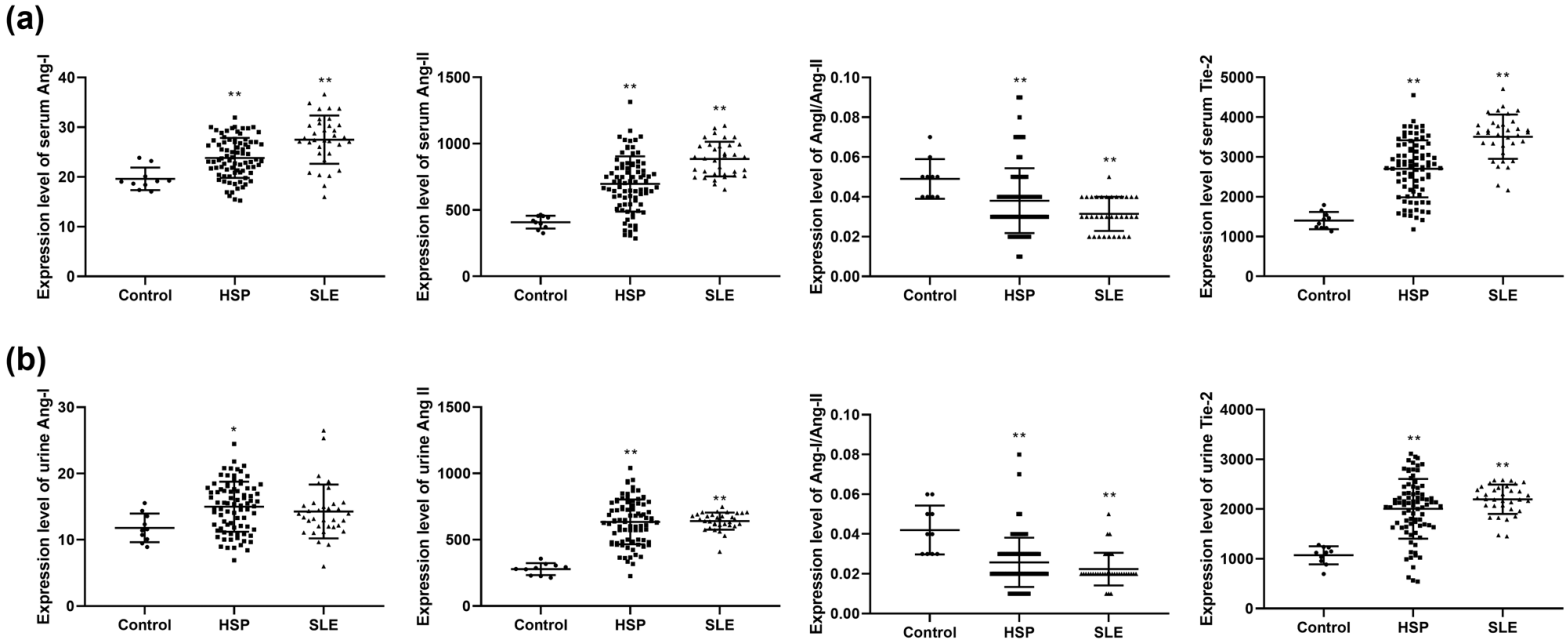
Expression levels of Ang-1, Ang-2, and Tie2 in serum and urine and Ang-1/Ang-2 values in control group, HSP group, and SLE group. (a) The levels of Ang-1, Ang-2, Ang-1/Ang-2, and Tie2 in serum of control, HSP, and SLE groups. (b) The levels of Ang-1, Ang-2, Ang-1/Ang-2, and Tie2 in urine of control, HSP, and SLE groups. Compared with control group, ** indicates *p* < 0.001; * indicates *p* < 0.05.

First, we examined the expression levels of Ang-1, Ang-2, and Tie2 in patients with HSP and pSLE (Table 1 and Figure 1). In serum samples, the expression levels of Ang-1, Ang-2, and Tie2 were elevated in the HSP and pSLE groups compared to those in the control group, while the Ang-1/Ang-2 expression level was decreased (*p* ≤ 0.001). In the urine samples, the Ang-2 and Tie2 expression levels were higher, and Ang-1/Ang-2 expression levels were lower in both the HSP and SLE groups compared to those in the control group (*p* < 0.001). Furthermore, Ang-1 expression levels were markedly increased in the HSP group compared to those in the control group (*p* = 0.038). However, no significant elevation was observed in the pSLE group (*p* > 0.05).

**Table 1:** Expression levels of Ang-1, Ang-2, and Tie2 in serum and urine and Ang-1/Ang-2 values of the control, HSP, and SLE groups

	Control (*n* = 10)	HSP (*n* = 82)	SLE (*n* = 34)	*F*/*H*	*p*
**Serum**					
Ang-1	19.62 ± 2.25	23.83 ± 4.02**	27.50 ± 4.85**	16.766	<0.001
Ang-2	408.52 ± 47.96	696.38 ± 208.47**	883.82 ± 130.85**	28.852	<0.001
Ang-1/Ang-2	0.05 (0.04, 0.05)	0.03 (0.03, 0.04)**	0.02 (0.02, 0.04)**	15.090	0.001
Tie2	1402.53 ± 215.49	2701.83 ± 715.65**	3507.51 ± 556.04**	44.033	<0.001
**Urine**					
Ang-1	11.80 ± 2.16	15.00 ± 3.78*	14.27 ± 4.05	3.345	0.038
Ang-2	278.36 ± 44.80	634.08 ± 168.68**	640.16 ± 64.62**	29.432	<0.001
Ang-1/Ang-2	0.04 (0.03, 0.05)	0.02 (0.02, 0.03)**	0.02 (0.02, 0.02)**	19.129	<0.001
Tie2	1071.35 ± 181.95	2006.36 ± 599.98**	2196.11 ± 295.39**	18.829	<0.001

Note: Compared with control group, ** indicates *p* < 0.001; * indicates *p* < 0.05.

### 3.3 Ang-1, Ang-2, and Tie2 levels in different types of HSP

**Figure 2:**
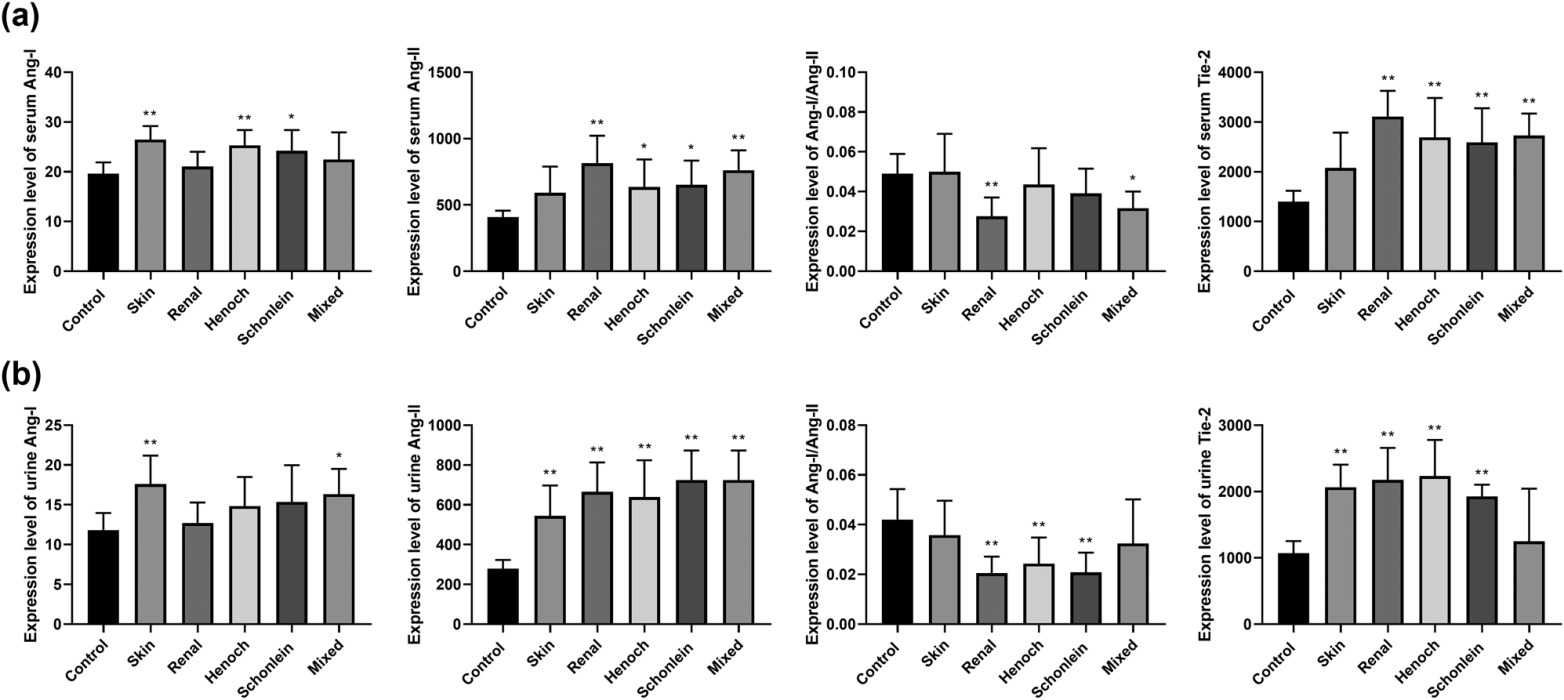
Expression levels of Ang-1, Ang-2, and Tie2 in serum and urine and Ang-1/Ang-2 values in different types of children with HSP. (a) Expression levels of Ang-1, Ang-2, Ang-1/Ang-2, and Tie2 in serum of control and different types of children with HSP groups. (b) Expression levels of Ang-1, Ang-2, Ang-1/Ang-2, and Tie2 in urine of control and different types of children with HSP groups. Compared with control group, ** indicates *p* < 0.001; * indicates *p* < 0.05.

Subsequently, the expression levels of Ang-1, Ang-2, and Tie2 in patients with different types of HSP were investigated (Table 2 and Figure 2). In the serum, compared to that in the control group, the Ang-1 expression level was higher in patients with skin, abdominal, and joint HSP (*p* < 0.05). The expression level of Ang-2 was elevated in cases of renal, abdominal, joint, and mixed HSP compared to the control group (*p* < 0.05). In addition, Ang-1/Ang-2 ratio was reduced in renal and mixed HSP. In the urine samples, the Ang-1 expression levels were higher in skin and mixed HSP compared with those in the control group (*p* < 0.05). The Ang-2 expression levels were increased in skin, renal, abdominal, joint, and mixed HSP compared to the control group (*p* < 0.05). In renal, abdominal, and joint HSP, the Ang-1/Ang-2 ratio was lower than that in the control group (*p* < 0.001). When compared to the control group, the Tie2 expression levels were increased in skin, renal, abdominal, and joint HSP (*p* < 0.001).

**Table 2:** Expression levels of Ang-1, Ang-2, and Tie2 in serum and urine and Ang-1/Ang-2 values in different types of children with HSP

	Control (*n* = 10)	Skin (*n* = 12)	Renal (*n* = 21)	Abdominal (*n* = 25)	Joint (*n* = 12)	Mixed (*n* = 12)	*F*/*H*	*p*
**Serum**								
Ang-1	19.62 ± 2.25	26.47 ± 2.70**	21.10 ± 2.93	25.31 ± 3.06**	24.26 ± 4.12*	22.49 ± 5.44	7.933	<0.001
Ang-2	408.52 ± 47.96	592.03 ± 197.15	815.59 ± 205.88**	635.96 ± 207.77*	652.54 ± 182.05^*^	761.83 ± 150.60**	7.785	<0.001
Ang-1/Ang-2	0.05 (0.04, 0.05)	0.05 (0.03, 0.07)	0.03 (0.02, 0.03)**	0.04 (0.03, 0.05)	0.04 (0.03, 0.05)	0.03 (0.02, 0.04)*	28.430	<0.001
Tie2	1402.53 ± 215.49	2077.58 ± 709.53	3112.76 ± 514.98**	2694.76 ± 789.67**	2593.42 ± 685.17**	2730.08 ± 440.85**	12.015	<0.001
**Urine**								
Ang-1	11.80 ± 2.16	17.62 ± 3.56**	12.70 ± 2.59	14.84 ± 3.65	15.37 ± 4.59	16.34 ± 3.17*	5.423	<0.001
Ang-2	278.36 ± 44.80	544.27 ± 153.23**	665.58 ± 147.17**	639.33 ± 184.66**	724.99 ± 148.02**	566.92 ± 160.16**	11.736	<0.001
Ang-1/Ang-2	0.04 (0.03, 0.05)	0.03 (0.03, 0.04)	0.02 (0.02, 0.02)**	0.02 (0.02, 0.03)**	0.02 (0.01, 0.03)**	0.03 (0.02, 0.04)	30.166	<0.001
Tie2	1071.35 ± 181.95	2067.03 ± 340.27**	2175.62 ± 483.38**	2234.01 ± 545.62**	1929.13 ± 175.07**	1252.42 ± 791.76	13.876	<0.001

Note: Compared with control group, ** indicates *p* < 0.001; * indicates *p* < 0.05.

### 3.4 Ang-1, Ang-2, and Tie2 levels in different types of pSLE

**Figure 3:**
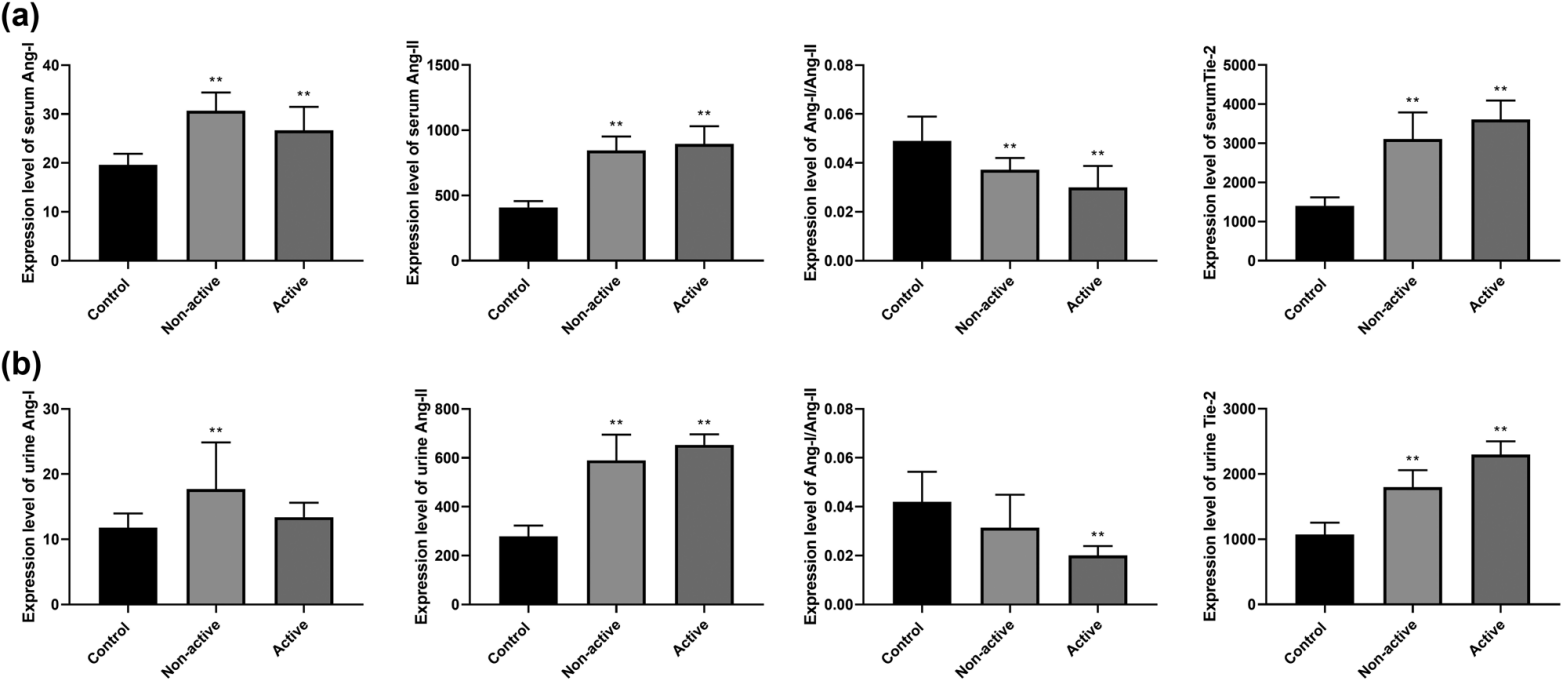
Expression levels of Ang-1, Ang-2, and Tie2 in serum and urine and Ang-1/Ang-2 values in different types of pSLE groups. (a) Expression levels of Ang-1, Ang-2, Ang-1/Ang-2, and Tie2 in serum of control and different types of pSLE. (b) Expression levels of Ang-1, Ang-2, Ang-1/Ang-2, and Tie2 in urine of control and different types of pSLE groups. Compared with control group, ** indicates *p* < 0.001; * indicates *p* < 0.05.

We then evaluated Ang-1, Ang-2, and Tie2 levels associated with different types of pSLE (Table 3 and Figure 3). In serum samples, compared to those in the control group, the expression levels of Ang-1, Ang-2, and Tie2 were increased in both active and non-active pSLE (*p* < 0.05). In the urine samples, the expression level of Ang-1 was elevated in patients with non-active pSLE compared to that in the control group (*p* < 0.001). In both active and non-active pSLE, the expression levels of Ang-2 and Tie2 were higher than that in the control group (*p* < 0.001).The Ang-1/Ang-2 ratio was reduced both in the serum and urine samples in active pSLE (*p* < 0.001).

**Table 3:** Expression levels of Ang-1, Ang-2, and Tie2 in serum and urine and Ang-1/Ang-2 values in different types of pSLE

	Control (*n* = 10)	Non-active (*n* = 7)	Active (*n* = 27)	*F*/*H*	*p*
**Serum**					
Ang-1	19.62 ± 2.25	30.68 ± 3.73**	26.67 ± 4.81**	15.959	<0.001
Ang-2	408.52 ± 47.96	843.96 ± 107.40**	894.16 ± 136.13**	63.096	<0.001
Ang-1/Ang-2	0.05 (0.04, 0.05)	0.04 (0.03, 0.04)	0.03 (0.02, 0.04)**	19.607	<0.001
Tie2	1402.53 ± 215.49	3107.94 ± 681.56**	3611.09 ± 480.72**	79.312	<0.001
**Urine**					
Ang-1	11.80 ± 2.16	17.72 ± 7.15**	13.38 ± 2.24	6.496	0.004
Ang-2	278.36 ± 44.80	589.51 ± 105.68**	653.29 ± 43.05**	159.067	<0.001
Ang-1/Ang-2	0.04 (0.03, 0.05)	0.03 (0.02, 0.04)	0.02 (0.02, 0.02)**	26.074	<0.001
Tie2	1071.35 ± 181.95	1797.51 ± 261.53**	2299.46 ± 202.72**	128.944	<0.001

Note: Compared with control group, ** indicates *p* < 0.001.

### 3.5 Ang-1, Ang-2, and Tie2 in serum and urine can be used as diagnostic biomarkers for HSP and pSLE

**Figure 4:**
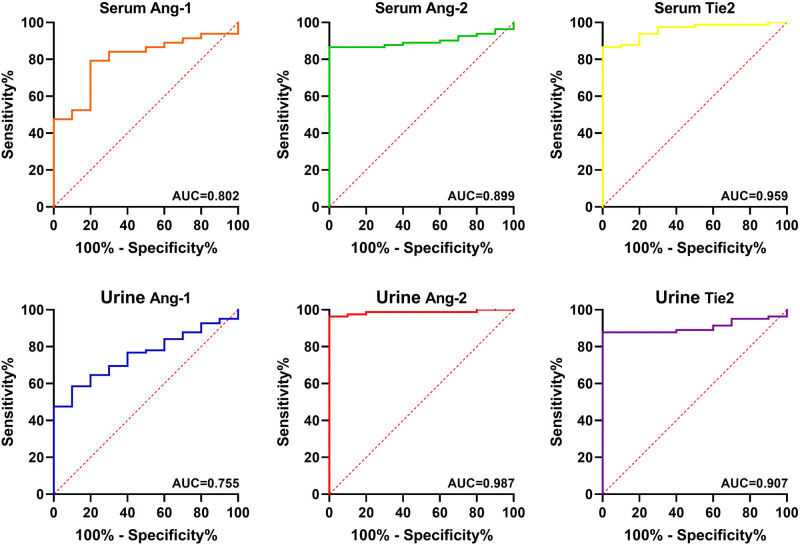
ROC curves of Ang-1, Ang-2, and Tie2 in serum and urine for the diagnosis of HSP.

**Figure 5:**
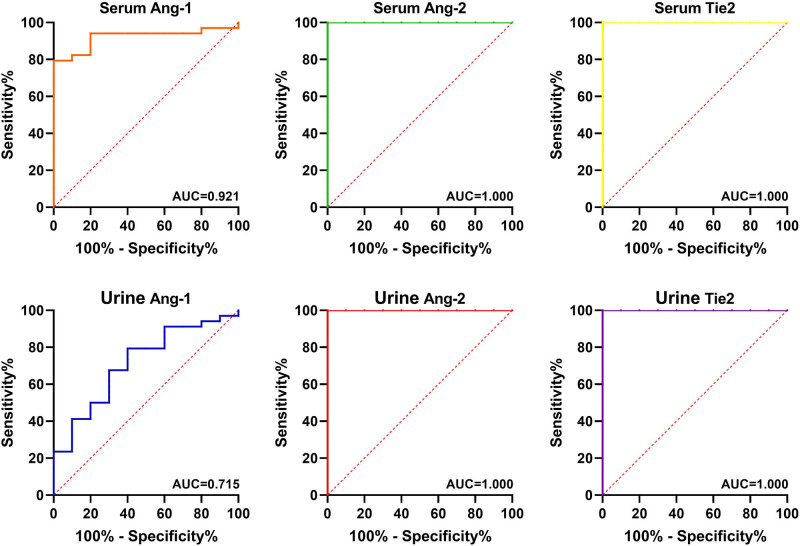
ROC curves of Ang-1, Ang-2, and Tie2 in serum and urine for the diagnosis of pSLE.

Then, we used ROC curves to examine whether the Ang-1, Ang-2, and Tie2 might be used as biomarkers to identify HSP or pSLE patients (Figures 4 and 5). The AUC values of Ang-1, Ang-2, and Tie2 in both, the serum and urine of HSP or pSLE patients were greater than 0.7, suggesting that they offer a high level of diagnostic accuracy in discriminating HSP from healthy tissue.

## 4 Discussion

HSP is the most frequently occurring small-vessel leukocytoclastic vasculitis in childhood, characterized by the predominant deposition of IgA-immune complexes in and around small blood vessels [28,29]. SLE is a chronic autoimmune disease with important correlations to cardiovascular disease and inflammation, impacting critical organs, such as central nervous system vasculitis and thrombotic microangiopathy [30–32]. Children with HSP and SLE often experience extensive diffuse organ involvement more often than adults do [33,34]. Diagnosing HSP and pSLE remains challenging due to the heterogeneous presentation of symptoms and signs, as well as the absence of specific diagnostic tests [35,36]. A diagnostic biomarker is a biological marker primarily used to detect and confirm a specific disease and identify individuals with a particular subtype [37]. ROC curves have facilitated a systematic approach to evaluate biomarkers [38]. In the present study, compared to the levels in the control group, the Ang-2 and Tie2 levels were significantly elevated in both the serum and urine of HSP and pSLE patients, while the Ang-1/Ang-2 ratio was reduced. Additionally, Ang-1 was highly expressed in the serum and urine of HSP patients and in the serum of pSLE patients. Furthermore, Ang-1, Ang-2, and Tie2 exhibited differential expression patterns across various types of HSP and pSLE. Ang-1, Ang-2, and Tie2 in the serum and urine may serve as potential biomarkers for HSP and pSLE, as indicated by the ROC curve analysis. Ang-1 and Ang-2 belong to a family of vascular growth factors that exert contrasting physiological effects on endothelial activation and dysfunction when combined with Tie2. Ang-1 binding stimulates vascular growth and stability, reduces inflammation, and supports endothelial cell survival [39,40]. In addition to its important role in angiogenesis and vascular stabilization, Ang-1 also maintains vascular permeability and lymphatic integrity [41]. Ang-2, often referred to as a Tie-2 antagonist, counteracts the stabilizing effect of Ang-1 by causing the loss of intercellular contact through autocrine signaling of the Tie-2 receptor [21]. Ang-2 binding promotes vascular activation, vascular inflammation, microvascular leakage, and neoangiogenesis [25,40]. Blood vessel growth, maintenance, leakiness, and instability depend on the local balance between Ang-1 and Ang-2 as well as other angiogenic agents [25]. Furthermore, Ang-1 has multiple anti-inflammatory properties, while Ang-2 promotes inflammatory responses [42,43]. The increase in Ang-2 and Tie2, but not in Ang-1 suggest a shift in the balance toward endothelial cell activation and inflammation [44]. In our investigation, the Ang-1/Ang-2 ratio was reduced in HSP and pSLE patients. A decrease in the Ang-1/Ang-2 ratio may promote the development of HSP and pSLE diseases. Moreover, a dysregulation of the balance between Ang-1 and Ang-2 can serve as a predictive factor for multiple diseases. The Ang-1/Ang-2 ratio is significantly elevated in diabetic retinopathy, and the difference in Ang-1/Ang-2 ratio precedes measurable changes in Ang-1 and Ang-2 individually, suggesting the Ang-1/Ang-2 ratio as a potential diagnostic biomarker [45]. Plasma Ang-2 levels are associated with cytokines in patients with sepsis, and the Ang-2/Ang-1 ratio is a reliable predictor of 28-day mortality in these individuals [46]. In children with septic shock, the Ang-1/Ang-2 ratio is reduced, and is associated with an increased use of vasoactive agents, prolonged intensive care unit stay, and correlation with the severity of illness score [47]. Therefore, we speculate that the Ang-1/Ang-2 ratio may serve as a biomarker of HSP and pSLE.

HSP can be classified into various types based on clinical traits. Skin HSP is an inflammatory disorder affecting the walls of dermal blood vessels and can cause a diverse range of clinical manifestations including urticarial lesions, plaques, papules, palpable purpura, ulcers, nodules, and livedo [48]. Renal HSP can occur in a considerable proportion of HSP patients, with rates as high as 30–50%, and children (1–3%) still experience renal insufficiency that can progress to end-stage renal failure [49,50]. The clinical features of abdominal HSP include diarrhea, abdominal pain, vomiting, gastrointestinal bleeding and blood in the stool, and even intussusception and intestinal perforation, which may be followed by intestinal obstruction and intussusception in severe cases [51,52]. Patients with joint-type HSP exhibit symptoms, such as swelling and pain in the joints [53]. Mixed HSP patients present with symptoms of more than two types of HSP. In our study, we found a significant reduction in the Ang-1/Ang-2 ratio in both urine and serum of patients with renal HSP. Renal HSP more frequently affects children and is the leading cause of secondary kidney diseases in pediatric patients [54]. Additionally, renal dysfunction is the main factor contributing to mortality in children with HSP [55]. Extensive IgA deposits have been identified in the renal vessels of HSP patients, and renal damage plays a pivotal role in their prognosis [56]. A decreased Ang-1/Ang-2 ratio may contribute to the development of nephropathy in HSP. Different HSPs are regulated by different genes [57]. For example, HMGB1 has an altered distribution in the lesional skin of HSP patients and may be an important mediator of endothelial inflammation through the induction of tumor necrosis factor-alpha and interleukin-6 production [57]. Serum galactose-deficient IgA1 levels are highly heritable in pediatric patients with renal-type HSP [58]. In the present study, we found that Ang-1, Ang-2, and Tie2 were differentially expressed in various types of HSP. pSLE is classified into two types, the non-active and active pSLE is regulated by multiple genes. In patients with active pSLE, the serum concentrations of VEGF, Tie2, and thrombomodulin are markedly elevated, whereas that of serum ADAMTS13 is reduced compared to the levels in individuals with inactive diseases [8]. In the present study, we identified distinct expression patterns of Ang-1, Ang-2, and Tie2 in different types of pSLE. Consequently, we hypothesized that Ang-1, Ang-2, and Tie2 may be used as biomarkers to distinguish between different types of HSP and pSLE.

Recently, an increasing number of diagnostic biomarkers for HSP and pSLE have been identified. The MCP-1 levels in the serum and urine were significantly elevated in SLE patients compared to those in healthy individuals, irrespective of the degree of disease activity and renal involvement, and it can be used as a biomarker for SLE [59]. In adult HSP patients, elevated VEGF-A and VEGFR-1 levels and the VEGFR-1/VEGF-A ratio in serum may serve as valuable indicators of inflammatory processes and vascular endothelial injury, and VEGFR-1 appears to be a particularly important marker in disease progression [60]. VEGF, Tie2, thrombomodulin, and ADAMTS13 are potent biomarkers for pSLE activity and organ involvement [8]. Immune complexes, anti-endothelial cell antibodies, and the pro-inflammatory effects of various cytokines can mediate vasculopathy in pSLE [61]. Furthermore, genes associated with endothelial dysregulation have been identified as highly effective biomarkers for the activity of pSLE and the extent of organ involvement in affected patients [8]. Thus, endothelial cell factors play an important role in HSP and pSLE patients.

In this study, it was found that Ang-1, Ang-2, and Tie2 in the serum and urine can serve as biomarkers for HSP and pSLE.

## 5 Conclusion

Ang-1/Ang-2 levels are reduced in HSP and pSLE patients. Ang-1, Ang-2, and Tie2 are promising biomarkers for HSP and pSLE. These biomarkers may prove valuable in elucidating vascular pathogeneses and in monitoring disease progression.
